# Drivers and perceived constraints on Dutch dairy farms to engage in disease prevention

**DOI:** 10.3389/fvets.2023.1124500

**Published:** 2023-03-30

**Authors:** Ruurd Jorritsma, Jantijn Swinkels, Tine van Werven, Nadia Lahaye, Merel Martena, Marijn Stok

**Affiliations:** ^1^Department Population Health Sciences, Faculty of Veterinary Medicine, Utrecht University, Utrecht, Netherlands; ^2^MSD Animal Health, Boxmeer, Netherlands; ^3^University Farm Animal Practice, Harmelen, Netherlands; ^4^Department of Interdisciplinary Social Science, Faculty of Social and Behavioral Sciences, Utrecht University, Utrecht, Netherlands

**Keywords:** disease prevention, drivers, constraints, dairy (cows), cattle, farmers' behavior

## Abstract

**Introduction:**

While prevention is increasingly important in the dairy sector, implementation of cost-effective preventive measures is often lacking. To increase the use of these measures and consequently improve animal welfare and reduce financial losses for farmers, it is necessary to know the drivers and constraints of farmers to engage in prevention.

**Methods:**

Therefore, we invited farmers to participate in an online questionnaire, which contained questions about their behavior toward either claw health or calf health. We used the theory form the Stage of Change model, COM-B, as well as the Theory of Planned Behavior to formulate our questions. We used the responses of 226 farmers in our analyses, who were equally distributed over the two groups of diseases.

**Results and discussion:**

We found that 63.5% of responding farmers were in the action phase or the maintenance phase to prevent claw diseases and even more (85.4%) to prevent calf diseases. The responses also suggest that many farmers have the knowledge and skills to implement preventive measures for both claw and calf diseases. The scores for social and physical opportunities for calf diseases were significantly higher than for claw diseases and all other COM-B components were also numerically higher for calf diseases. This suggests that farmers' perception of taking preventive measures against claw diseases is more difficult than taking preventive measures against calf disease. The automation of preventive behavior scored relatively low for both groups of diseases, which suggests that farmers may need reminders to persist in their activities and support to create habitual prevention behaviors. From these results, we concluded that creating social norms, supporting discussions among farmers, and using environmental adaptations may result in more preventive behavior.

## Introduction

Prevention of disease is key in the dairy sector. It reduces for example the hazard that milk or meat contains traces of antibiotics or other drugs, but it is also beneficial for animal welfare. Moreover, it is known that the prevention of diseases reduces the workload for farmers, additional costs for treatments, and therefore results in higher profits for the dairy farmer (1).

There are many effective measures for the prevention of important diseases that occur on dairy farms, such as mastitis and lameness. Preventive measures are usually classified into three categories. These categories are different with respect to whether and to which degree a disease did already occur or not. In tertiary prevention, the aim is to soften the impact of a clinical disease and prevent the occurrence of new cases. In secondary prevention, the aim is to prevent that subclinical disease becomes clinical. Primary prevention may be regarded as the highest level as the intention is to prevent new diseases in healthy individuals, by decreasing risk factors for disease. Thus, in primary prevention, preventive measures are taken before problems occur and therefore these measures are not serving a direct cause. In many cases, diseases such as lameness, mastitis, gastro-intestinal diseases and respiratory diseases, are endemic in a herd. Thus, farmers are usually considering tertiary prevention strategies.

Some of these preventive strategies were found beneficial from an economic point of view (2, 3). It is also known that many farmers are, despite estimated economic advantages, not always inclined to introduce and continue to adhere to such preventive strategies once they started one (4). This does not mean that economics are not important, as farmers appeared to be sensitive to prevent losses and penalties rather than to increase gains by improving health (5). Studies on disease prevention in the human domain also demonstrated that economic effects do not always prevail. In general, people preferred disease prevention from an economic point of view, but rather preferred treatment in case of perceived urgency (6). Moreover, adherence to preventive strategies is, among others, supposed to be hampered by the long time interval between the preventive intervention and its effect, by the unclear link between an intervention and its effect, and by the uncertainty that the intervention will have an effect (6).

As economic arguments appeared at least to be not the only motivators, others have explored the drivers and barriers for the implementation of preventive measures by farmers. In a study by Brennan et al. (7), farmers were found hesitant to implement prevention and control measures in case they were not convinced about the quality of diagnostic tests, effectiveness, or time efficiency. Bruijnis et al. showed that, apart from labor efficiency, also the long interval between the intervention and clinical improvement may withhold some farmers to implement preventive measures to improve foot health (8).

We are aware of studies on the prevention of zoonoses on farms and on the implementation of biosecurity measures at the level of the farm or the region (9–11). While these publications did in fact address primary prevention, they did not address the prevention of a specific disease in a herd. Instead, they focused on general measures to prevent in particular the introduction of diseases into a herd and not on the prevention of diseases caused by e.g., environmental pathogens.

While others reported an increasing number of reports on farmers' motivators and barriers with respect to disease control, they concluded that these studies frequently lack underpinning or explicit theory and gave some suggestions for improvement (12). One of the mentioned and generally accepted useful models is the transtheoretical model (13). It contains different stages that people move through in the process of (preparing for) behavior change. The recognized stages are precontemplation (no serious intention to change behavior), contemplation (there is a serious intention to change behavior in due time, but not right now); preparation (there is a serious intention to change behavior in the short term), action (behavior change is currently happening) and maintenance (behavior change has already occurred and is being maintained).

A second well-established model of behavior change is the COM-B model (14), which addresses the issue of whether people have the capabilities (knowledge, skills and physical abilities), opportunities (presence of physical and social environment to facilitate engaging in the behavior), and motivation (motivated and habituated) to perform a behavior. Third, the widely applied theory of planned behavior posits that people's intention to engage in a behavior is guided by attitudes regarding the behavior, subjective norms of how others behave and expect those around them to behave, and perceived behavioral control or the extent to which people believe they have personal control over the behavior (15).

We decided to use these three models to explore the drivers and perceived constraints of Dutch dairy farmers to engage in primary prevention toward calf diseases and claw diseases using an online questionnaire and to identify whether these diseases are approached similar.

## Materials and methods

### Procedure

A convenience sample of Dutch dairy farmers, consisting of all clients of 14 veterinary clinics (~2,000 farmers in total) collaborating in the Association “Vereniging Kernpraktijken Rundvee” was invited to participate in an online survey about primary prevention behavior. Farmers received an e-mail from their own veterinary clinic explaining the general purpose of the survey. The e-mail contained a link to the survey (created using Qualtrics XM^®^). When clicked, this link led to a webpage with additional information about the study and a checkbox to indicate informed consent. Farmers were then asked to provide some general information about their farm and to indicate which veterinary clinic they belonged to. They were subsequently routed, randomized within each clinic, to either the survey about claw diseases or calf diseases. The survey included close-ended questions probing the relevant psychological constructs from the three guiding frameworks, open-ended questions to allow respondents to provide additional in-depth information about either the prevention of claw or calf diseases, and questions about specific prevention behaviors. Participation in the survey was voluntary and anonymous. Respondents were not paid for their participation, but all participants who completed the entire survey received afterwards either a claw knife or a colostrum testing tool as a thank you when they presented themselves at their veterinary clinic.

### Participants

A total of 323 farmers clicked the link to the survey, but 81 respondents (25%) did not advance beyond the provision of informed consent or proceeded at another moment, and 7 respondents (2%) only provided general information about their farm and then exited the survey. Nine farmers indicated not having any dry or lactating cows on their farm, but only youngstock. These farmers were excluded from the analyses. This left a sample of 226 farmers who proceeded to the actual survey, with similar numbers routed to the survey about claw diseases (*N* = 117) and calf diseases (*N* = 109). Of these 226 respondents, 197 completed the entire survey. The other 29 respondents filled out only a part of the survey; these respondents are included in analyses on the items they did complete.

### Materials

The full survey (in Dutch) is on request online available *via* https://doi.org/10.17026/dans-xsm-xqfk.

#### Stage of change

Farmers were asked to indicate which stage of change about taking measures promoting either claw or calf health, respectively, best described them. Answer options ranged from (15) “I don't often think about ways I can prevent claw / calf health problems” (i.e., precontemplation) to (8) “I have been taking various measures to prevent claw / calf health problems for a long time already, and do not see how I could further improve on this” (i.e., maintenance).

#### COM-B

Farmers were asked to indicate (on a scale ranging from 1 “completely disagree”to 7 “completely agree”) to which extent they agreed with six statements probing the six components of the COM-B model:

1. physical capability (“I am physically capable of taking measures to prevent claw / calf health problems: I do not have any physical issues that limit my capabilities, or I ensure that someone else does this work”);
2. psychological capability (“I know which measures I can take to prevent claw / calf health problems to start: I have sufficient knowledge about this”);
3. social opportunity (“employees, advisors and colleagues motivate me to do everything I can to prevent claw/calf health problems”);
4. physical opportunity (“I have good equipment, good facilities, and enough time to take the measures I want to take to prevent claw/calf health problems”);
5. reflective motivation (“I find it important to prevent claw/calf health problems from developing”);
6. automatic motivation (“Taking measures to prevent claw/calf health problems has become routine for me, it goes more or less automatically”).

#### Theory of planned behavior

To explore Theory of Planned Behavior-related constructs, three specific preventive behavior measures were selected both for claw and calf health. For the farmers routed to the survey on claw health, these measures were keeping floors dry and clean; preventive claw trimming; and preventing overcrowding. For farmers routed to the survey on calf health, these specific measures were feeding colostrum within 2 h after birth; using a separate maternity unit; and cleaning and disinfecting the calf hutches after every use. For each of these topics, the following constructs were then assessed:

1. Attitude was measured by asking respondents to select how (un)necessary (scale ranging from 1 “completely unnecessary” to 7 “completely necessary”) and how (un)important (scale ranging from 1 “completely unimportant” to 7 “completely important”) they found each behavioral measure. An average attitude measure was computed for each behavior (all Pearson's *r*'s > *0.8*5, all *p*'s < 0.001).
2. Subjective norms were assessed with two items per behavior, probing respondents' agreement with statements others considering each measure important (descriptive norm, e.g. “my employees and other cattle farmers I have frequent contact with find it important to feed colostrum within 2 h of birth”) and statements about others engaging in each measure (injunctive norm, e.g., “most cattle farmers I know prevent overcrowding in their stables”). Subjective norms were assessed on a scale of 1 (“completely disagree”) to 7 (“completely agree”). An average subjective norm measure was computed for each behavior (all Pearson's *r*'s ≥ 0.50, all *p*'s < 0.001).
3. Perceived behavioral control was assessed with one item per measure, which asked respondents to indicate to which extent they agreed that performing the respective prevention behavior is something they could always take care of (e.g., “I can ensure that calf hutches are cleaned and disinfected after every use; scale of 1, ‘completely disagree' to 7, ‘completely agree”').
4. Intention to perform each behavioral measure was assessed by asking respondents to which extent they agreed with statements about wanting to implement each measure (e.g., “I want the floors to always be dry and clean”; (scale of 1, “completely disagree” to 7, “completely agree”).

*Most important prevention measures*. An open-ended question asked farmers to indicate what they thought were the two most effective prevention measures to improve claw or calf health, respectively.

*Most needed resources*. Finally, a multiple-choice question probed which resources farmers were most in need of in order to take more prevention measures: “What do you need most to be able to take more measures that can prevent claw / calf health problems?” Answer options were “more time”; “more manpower”; “more money”; “more knowledge”, and “other, namely” with an open-answer field provided. Respondents could select as many answer options as they wanted.

### Statistical analyses

For stage of change, frequencies were computed. For COM-B variables, means and standard deviations were computed and for each variable, significance tests were performed probing differences between claw and calf health prevention behavior. For TPB variables, means and standard deviations were computed for each of the six behaviors. In addition, multiple regression analyses were performed to determine to what extent attitude, subjective norm and perceived behavioral control were related to intention to engage in each of the six specific behaviors, controlling for 305 day production, herd size, and available labor power. Categorizations were made of the open answers regarding the most important prevention measures, and frequencies were computed for the most needed resources indicated by respondents in the corresponding multiple choice question.

## Results

### Descriptive statistics

The average 305 day production was *M* = 9,449 kg/yr (*SD* = 1,433), the average herd size of the farms participating was *M* = 129 cows (*SD* = 96, range 14–800) and the average labor power was *M* = 1.73 FTE (*SD* = 1.0). These results did not differ between farmers completing the claw vs. calf health surveys [all *Fs* (1, 222) < 2.50, all *p*s > 0.120], indicating successful randomization. The datasets are on request online available at https://doi.org/10.17026/dans-xsm-xqfk.

### Stage of change

Most dairy farmers were in the action and maintenance stages of change (see Table 1), indicating that some prevention behaviors are typically already being carried out and often also stably maintained. However, there were substantial differences in frequencies of stages of change between prevention behaviors regarding claw health vs. calf health. A chi-square test indicated that these differences are statistically significant [*X*^2^ = (4, *N* = 226) = 11.44, *p* = 0.022]. Compared to calf health problem prevention, farmers are more often in the contemplation and preparation phases, and less often in the maintenance phase, when it regards claw health problem prevention.

**Table 1:** Frequencies of stages of change for claw and calf health problem prevention behaviors.

	**Claw disease prevention (*N* = 117)**	**Calf disease prevention (*N* = 109)**
Precontemplation	5.1% (*N* = 6)	5.5% (*N* = 6)
Contemplation	12.8% (*N* = 15)	8.3% (*N* = 9)
Preparation	8.5% (*N* = 10)	0.9% (*N* = 1)
Action	43.6% (*N* = 51)	40.4% (*N* = 44)
Maintenance	29.9% (*N* = 35)	45.0% (*N* = 49)

### COM-B

Reflective motivation was very high for prevention behaviors for both types of health problems and highest of all COM-B components. This was followed by physical capability and psychological capability, which were both also rather high. Social opportunity was lowest for both types of prevention behaviors, although still slightly above the mid-point of the scale, meaning that respondents reported low to moderate social opportunities to engage in the behaviors. Scores for physical opportunity and automatic motivation fell in between the aforementioned components for both types of behavior, showing moderate physical opportunity and automatic motivation for engaging in the prevention behaviors. Means of the various COM-B components differed statistically both for claw health problem prevention [*F* (5, 105) = 27.26, *p* < 0.001] and for calf health problem prevention [*F* (5, 100) = 21.86, *p* < 0.001]; Table 2 provides exact information about which components differed from each other.

**Table 2:** Means and standard deviations for COM-B components for claw and calf health problem prevention behaviors.

	**Claw health problem prevention (*N* = 110)**	**Calf health problem prevention (*N* = 105)**	**Difference claw-calf (two-sided significance testing)**
Physical capability	*M* = 5.46^c^, *SD* = 1.60	*M* = 5.87^e^, *SD* = 1.50	*T* (213) = −1.91, *p* = 0.058
Psychological capability	*M* = 5.38^c^, *SD* = 1.27	*M* = 5.49^bcd^, *SD* = 1.44	*T* (213) = −0.56, *p* = 0.574
Social opportunity	*M* = 4.57^a^, *SD* = 1.37	*M* = 5.02^ab^, *SD* = 1.49	*T* (213) = −2.29, *p* = 0.023
Physical opportunity	*M* = 4.83^ab^, *SD* = 1.65	*M* = 5.36^bcd^, *SD* = 1.45	*T* (213) = −2.52, *p* = 0.012
Reflective motivation	*M* = 6.11^d^, *SD* = 1.42	*M* = 6.29^f^, *SD* = 1.49	*T* (213) = −0.89, *p* = 0.374
Automatic motivation	*M* = 5.00^b^, *SD* = 1.54	*M* = 5.27^abcd^, *SD* = 1.54	*T* (213) = −1.27, *p* = 0.206

Means with different superscripts within the same column differ significantly at p < 0.05.

Comparing the COM-B components between the two types of health problems showed that farmers saw more opportunities, both social and physical, to engage in the selected prevention behaviors for calf health than for claw health. No differences were found between capabilities and motivations to engage in prevention behaviors for either type of health problems.

### Theory of planned behavior

Results (see Tables 3, 4) showed that dairy farmers viewed prevention behaviors mostly highly important and necessary, although there are considerable differences between the behaviors (with lowest attitude found for using a separate maternity unit). Subjective norms scored were considerably lower, however, although still above the midpoint for all six behaviors. Farmers thus perceive moderate descriptive and injunctive norms regarding prevention behaviors for claw and calf health. There were large differences between the behaviors with regard to the extent to which farmers perceive behavioral control; while perceived behavioral control was very high for preventive claw trimming, for example, it was much lower for using a separate maternity unit. Finally, intention to engage in each of the prevention behaviors was moderately high, with scores for all six behaviors ranging between 5 (slightly agree) to 6 (agree).

**Table 3:** Means and standard deviations for TPB components for the three specific behaviors related to claw health.

	**Attitude**	**Subjective norm**	**Perceived behavioral control**	**Intention**
Keeping floors dry and clean (*N* = 107)	*M* = 5.87^a^, *SD* = 1.37	*M* = 5.01^a^, *SD* = 1.13	*M* = 5.00^a^, *SD* = 1.68	*M* = 5.51^a^, *SD* = 1.54
Preventive claw trimming (*N* = 107)	*M* = 5.87^a^, *SD* = 1.59	*M* = 5.38^b^, *SD* = 1.10	*M* = 6.13^b^, *SD* = 1.13	*M* = 5.82^a^, *SD* = 1.72
Preventing overcrowding (*N* = 105)	*M* = 5.74^a^, *SD* = 1.25	*M* = 4.83^a^, *SD* = 1.23	*M* = 5.71^c^, *SD* = 1.65	*M* = 5.69^a^, *SD* = 1.57

Means with different superscripts within the same column differ significantly at p < 0.05.

**Table 4:** Means and standard deviations for TPB components for the three specific behaviors related to calf health.

	**Attitude**	**Subjective norm**	**Perceived behavioral control**	**Intention**
Feeding colostrum within 2 h of birth (*N* = 100)	*M* = 6.02^a^, *SD* = 1.16	*M* = 4.70^a^, *SD* = 1.16	*M* = 5.08^a^, *SD* = 1.75	*M* = 5.08^a^, *SD* = 1.84
Cleaning and disinfecting calf hutches after every use (*N* = 96)	*M* = 5.90^a^, *SD* = 1.26	*M* = 5.08^b^, *SD* = 1.10	*M* = 5.78^b^, *SD* = 1.42	*M* = 5.60^b^, *SD* = 1.66
Using a separate maternity unit (*N* = 95)	*M* = 5.47^b^, *SD* = 1.55	*M* = 4.76^a^, *SD* = 1.24	*M* = 4.75^a^, *SD* = 2.21	*M* = 5.28^ab^, *SD* = 1.92

Means with different superscripts within the same column differ significantly at p < 0.05.

Regression analyses (see Table 5) showed that 305 day production, herd size, and available labor power were mostly unrelated to intentions to engage in the prevention behaviors. On the contrary, attitude and perceived behavioral control were consistently and strongly related to intentions to engage in all specific prevention behaviors. Findings for subjective norms were less consistent; an association was found to intention to feed colostrum within 2 h of birth and intention to clean and disinfect calf hutches after every use, but not to intentions to engage in the other four prevention behaviors.

**Table 5:** Regression analyses on Theory of Planned Behavior constructs for all six specific prevention behaviors.

	**Keeping floors dry and clean (*N* = 107)**	**Preventive claw trimming (*N* = 107)**	**Preventing overcrowding (*N* = 105)**	**Feeding colostrum within 2 h of birth (*N* = 100)**	**Cleaning and disinfecting calf hutches after every use (*N* = 96)**	**Using a separate maternity unit (*N* = 95)**
305 day production	*t* = 1.41, *B* = 0.00, β = 0.10	*t* = 0.10, *B* = 0.00, β = 0.01	*t* = −1.51, *B* = −0.00, β = −0.01	*t* = −0.12, *B* = −0.00, β = −0.01	*t* = 0.04, *B* = 0.00, β = 0.00	*t* = −0.74, *B* = −0.00, β = −0.05
Herd size	*t* = −0.64, *B* = −0.00, β = 0.10	*t* = 1.50, *B* = 0.00, β = 0.15	*t* = −2.00^*^, *B* = −0.00, β = −0.27	*t* = −1.78^†^, *B* = −0.01, β = −0.14	*t* = 0.70, *B* = 0.00, β = 0.05	*t* = −0.07, *B* = 0.00, β = −0.01
Labor power	*t* = 0.70, *B* = 0.15 β = 0.10	*t* = −1.69^†^, *B* = −0.28, β = −0.17	*t* = 1.66, *B* = 0.33, β = 0.23	*t* = 0.86, *B* = 0.19, β = 0.07	*t* = 1.42, *B* = 0.23, β = 0.09	*t* = 1.61, *B* = 0.37, β = 0.13
Attitude	*t* = 5.99^***^, *B* = 0.52, β = 0.46	*t* = 10.88^***^, *B* = 0.70, β = 0.65,	*t* = 5.84^***^, *B* = 0.58, β = 0.46	*t* = 3.85^***^, *B* = 0.46, β = 0.29	*t* = 5.84^***^, *B* = 0.53, β = 0.40	*t* = 4.38^***^, *B* = 0.44, β = 0.36
Subjective norm	*t* = 1.27, *B* = 0.13, β = 0.09	*t* = 1.46, *B* = 0.13, β = 0.09	*t* = 1.37, *B* = 0.12, β = 0.09	*t* = 4.36^***^, *B* = 0.51, β = 0.32	*t* = 2.64^*^, *B* = 0.25, β = 0.16	*t* =1.38, *B* = 0.17, β = 0.11
Perceived behavior control	*t* = 4.09^***^, β = 0.34	*t* = 4.76^***^, *B* = 0.45, β = 0.30	*t* = 4.92^***^, *B* = 0.36, β = 0.38	*t* = 4.63^***^, *B* = 0.39, β = 0.37	*t* = 6.28^***^, *B* = 0.53, β = 0.45	*t* = 5.04^***^, *B* = 0.39, β = 0.45
Model statistics	*R*^2^ = 0.54, *F (*6,100) = 21.61^***^	*R*^2^ = 0.78, *F* (6,100) = 65.14^***^	*R*^2^ = 0.64, *F* (6,98) = 32.12^***^	*R*^2^ = 0.62, *F* (6,92) = 27.96^***^	*R*^2^ = 0.75, *F* (6,88) = 47.31^***^	*R*^2^ = 0.62, *F* (6,87) = 25.74^***^

^†^ indicates p < 0.10, ^*^ indicates p < 0.05, ^***^ indicates p < 0.001.

### Most important prevention measures

A total of 103 farmers indicated what they considered to be either the two (*N* = 102) or one (*N* = 1) most important prevention measures for claw health; 84 farmers indicated either two (*N* = 74) or one (*N* = 10) prevention measure that they considered most important for calf health. A categorization of the measures mentioned is provided in Table 6 and shows a reasonable convergence between farmers regarding the most important prevention measures. For claw disease prevention, improving floors is the most frequently mentioned measure, followed by optimizing footbaths, feed adjustments and improving regular claw trimming. For calf disease prevention, improving the housing of calves and optimizing colostrum feeding are most frequently mentioned, followed by improving general hygiene and biosecurity and feeding calf and dam.

**Table 6:** Most important prevention measures according to the respondents.

**Claw disease prevention**	**Calf disease prevention**
Improve floor (23%)	Improve housing calves (30%)
Optimize footbath (17%)	Optimize colostrum feeding (20%)
Feed adjustments (17%)	Improve general hygiene and biosecurity (12%)
Improve regular claw trimming (16%)	Feeding calf and dam (10%)
Early detection and treatment (6%)	Cleaning and/or disinfecting facilities calf (6%)
Cubicle bedding (5%)	Create or optimize hygiene maternity pen (6%)
Increase time on pasture (3%)	Vaccinations and treatments (6%)
Improve walking path outside (2%)	Other (9%)
Selection and breeding (2%)	
Other (8%)	

### Most needed resources

There were 97 farmers who indicated most needed resources for being able to take more prevention measures promoting claw health (with *N* = 60 indicating one resource, *N* = 32 farmers indicating two resources; and 5 farmers indicating three resources); 83 farmers indicated most needed resources for being able to take more prevention measures promoting calf health (with *N* = 52 indicating one resource, *N* = 25 farmers indicating two resources; five farmers indicating three resources; and *N* = 1 farmer indicating four resources). To facilitate taking more claw health prevention measures, farmers mostly indicated needing more time (29%), more money (28%), and more knowledge (24%); to facilitate taking more calf health prevention measures, farmers mostly indicated needing more time (35%) and more manpower (31%; see Table 7 for all frequencies). Results show that across both behaviors, time is the most crucial resource for engaging in more prevention behavior. However, additional most necessary resources were different for both behaviors (money and knowledge for claw health vs. manpower for calf health). Farmers could also indicate that they considered “other” resources to be most needed and were then asked to further specify which resources these were. Analysis of these open answers did not yield any clear categories that were mentioned by more than one farmer.

**Table 7:** Most needed resources for claw and calf health according to the respondents.

	**Claw diseases**	**Calf diseases**
More time	40 (29%)	42 (35%)
More manpower	7 (5%)	37 (31%)
More money	39 (28%)	10 (8%)
More knowledge	33 (24%)	21 (17%)
Other	20 (14%)	11 (9%)

## Discussion

It is generally known that the healthcare for, in particular, food animals continues to shift away from treatments of diseased animals toward interventions that prevent animals from acquiring a disease. While there is already a lot of attention for the efficacy of prevention strategies for many diseases, the number of publications on how to implement or improve the use of known preventive strategies by dairy farmers is in our opinion rather limited. This is disappointing in our opinion, as we feel that it is possible to make a huge potential improvement in animal welfare when all existing knowledge would be applied in practice. The increasing but still relatively limited attention for the implementation of preventive interventions seems even more out of balance considering that differences in e.g., economic circumstances, education levels, and culture will affect the mutual importance of specific drivers and barriers. Replication of behavioral studies across various settings is also necessary before insights from such behavioral studies can be implemented in interventions.

We agree with Biesheuvel et al. (12) that it is necessary to use theory driven studies to study how to improve the use of preventive interventions and therefore used three well-established and empirically tested models of behavior change to identify the drivers and barriers for preventive strategies of two different health issues. We rather subjectively selected claw diseases and calf diseases because we thought that they have an important impact on animal welfare and also because they likely affect the societal acceptance of the industry.

We restricted the number of preventive options and they therefore do not completely cover all specific underlying diseases. By doing so, we limited the number of questions and consequently the time to complete the questionnaire, which was in our view important to attract a sufficient number of respondents.

We acknowledge that the responding farmers in our study are not necessarily representative for the Netherlands, as they were somehow motivated to participate in the study and also because they were part of a convenience sample of clients from collaborating veterinary practices. Moreover, the results of the surveys are based on self-reported data and should be replicated both with in-depth interviews as well as studies including behavioral measures for further validation. Yet, our findings provide important initial insights into specific barriers and facilitators for prevention behaviors for claw and calf health, which can be used for the development of targeted interventions.

Our results suggest that many dairy farmers in the study are well aware of the importance of preventive measures as 63.5% of the farmers were in the action or maintenance phase to promote claw health and even more (85.4%) to promote calf health. These percentages are higher than those reported on the implementation of zoonotic control programs or the introduction of biosecurity (9, 10) and suggest that veterinary consultants do not always need to convince farmers about the importance of taking preventive measures. This might be due to the more frequent occurrence of the health issues in our study, which means that farmers will experience that their preventive interventions are soon effective.

While we found that adoption of prevention was relatively high, there is also still a considerable number of farmers that were not in the action or maintenance phase. Moreover, the results indicate that the level of prevention may differ between specific diseases. The responses of the farmers also suggest that many of them have the knowledge and skills to implement preventive measures for both claw health and calf health. Given the results, we think that it is valuable for veterinary consultants to check whether or not they need to discuss the need to implement preventive measures and to be aware of the fact that the majority of the farmers, but not all of them, have the capabilities to implement preventive strategies.

Interestingly, we found significantly higher scores of social and physical opportunities for calf diseases than for claw diseases. As all other COM-B components are also numerically higher for calf diseases, we think that farmers face more barriers in the prevention of claw diseases compared to the prevention of calf diseases. Given the answers in the open questions, farmers need in particular more knowledge and money for the prevention of claw diseases, whereas they need more time and manpower for the prevention of calf diseases. We may summarize that farmers perceive that, overall, taking preventive measures against claw diseases is more difficult than taking preventive measures against calf disease. Similar differences might occur with respect to other diseases.

For both claw diseases and calf diseases, the automation of preventive behavior scored relatively low, which suggests that farmers may need reminders to persist in their activities and support in the creation of habitual prevention behaviors. We suggest that veterinary consultants could give these reminders and support to the farmers.

Farmers also perceive limited environmental support for their preventive behavior, as evidenced by the fact that physical and social opportunities were clearly scored lower than other COM-B components. This suggests that creating social norms, supporting discussions among farmers, and using environmental adaptations to make preventive behaviors easier may result in more preventive behavior. We suggest advisors to create discussion or working groups with farmers, other advisors, and maybe the general public to exchange views and opinions. While regression analyses showed that perceived social norms were not strongly associated with preventive behavior in this study, previous research has shown that the effects of social norms remain frequently unnoticed (16). This would mean that self-report measures are not well suited to capture the effects of social norms, meaning that follow-up studies including more behavioral measures are necessary.

It is promising that many farmers have adopted the idea that prevention is important and are already engaging in various preventive behaviors. Yet, results show that there is enough room for improvement for in particular the prevention of claw diseases. This study also suggests that interventions targeting the environment (both physical and social) may be most effective to improve prevention, as there is still much room for improvement in this domain. Compared to the prevention of calf diseases, farmers seem to have more difficulties in the prevention of claw diseases and indicate that they need more knowledge and money for this.

## Data availability statement

The datasets presented in this study can be found in online repositories. The names of the repository/repositories and accession number(s) can be found below: DANS https://doi.org/10.17026/dans-xsm-xqfk.

## Ethics statement

The studies involving human participants were reviewed and approved by Ethics Review Board of the Faculty of Social and Behavioral Sciences of Utrecht University (#20-539). The patients/participants provided their written informed consent to participate in this study.

## Author contributions

RJ organized the project and supervised NL and MM, who were veterinary students when they performed the project, performed the study, and wrote the first draft of the manuscript. JS initiated and facilitated the study. TW contacted the veterinary clinics and edited the questions. MS designed the survey, analyzed the data, and also supervised NL and MM. RJ and MS wrote the final manuscript. NL and MM made comments, have read the final version, and agreed upon its content. All authors contributed to the article and approved the submitted version.
